# RETRACTION: Mechanism of Zuogui Pill Enhancing Ovarian Function and Skin Elastic Repair in Premature Aging Rats Based on Artificial Intelligence Medical Image Analysis

**DOI:** 10.1111/srt.70156

**Published:** 2025-01-07

X. Zhang, F. Wang, X. Zhu, L. Xu, L. Qin, W. Xu, and B. Fan, “Mechanism of Zuogui Pill Enhancing Ovarian Function and Skin Elastic Repair in Premature Aging Rats Based on Artificial Intelligence Medical Image Analysis,” *Skin Research and Technology* 30, no. 9 (2024): e70050, https://doi.org/10.1111/srt.70050.

The above article, published online on 9 September 2024 in Wiley Online Library (wileyonlinelibrary.com), has been retracted by agreement between *Skin Research and Technology*; and John Wiley & Sons Ltd. The article was submitted as part of a guest‐edited special issue. Following publication, it has come to the attention of the journal that the article was accepted solely on the basis of compromised editorial handling and peer review processes. Furthermore, the article deviates from its stated aims, and its conclusions are not supported by the presented data.

Therefore, the decision to retract this article was taken.

